# Memory-efficient, accelerated protein interaction inference with blocked, multi-GPU D-SCRIPT

**DOI:** 10.1093/bioinformatics/btaf564

**Published:** 2025-10-11

**Authors:** Daniel E Schäffer, Samuel Sledzieski, Lenore Cowen, Bonnie Berger

**Affiliations:** Computer Science and Artificial Intelligence Laboratory, Massachusetts Institute of Technology, Cambridge, MA 02139, United States; Computational and Systems Biology Program, Massachusetts Institute of Technology, Cambridge, MA 02139, United States; Center for Computational Biology, Flatiron Institute, New York, NY 10010, United States; Department of Computer Science, Tufts University, Medford, MA 02155, United States; Computer Science and Artificial Intelligence Laboratory, Massachusetts Institute of Technology, Cambridge, MA 02139, United States; Department of Mathematics, Massachusetts Institute of Technology, Cambridge, MA 02139, United States

## Abstract

**Summary:**

D-SCRIPT is a powerful tool for high-throughput inference of protein–protein interactions (PPIs), but it is expensive in time and memory to infer all PPIs for network-/proteome-level analyses. We introduce D-SCRIPT with blocked multi-GPU parallel inference, which substantially reduces memory usage across tasks and computational systems (13.8× for a representative large proteome) and enables multi-GPU parallelism.

**Availability and implementation:**

Blocked multi-GPU parallel inference has been integrated into the main D-SCRIPT package, available at https://github.com/samsledje/D-SCRIPT. An archived version of the code at time of submission can be found at https://doi.org/10.5281/zenodo.16325182.

## 1 Introduction

Rapid progress in sequencing has led to the availability of large numbers of protein sequences for many organisms across the tree of life, but experimentally validated protein–protein interaction (PPI) data are still labor-intensive to produce and hence available at scale for only a handful of model organisms. To bridge this gap, we previously introduced D-SCRIPT (Sledzieski *et al.* 2021), a deep learning method that predicts PPIs from protein sequences using protein language models. D-SCRIPT has become widely used to predict PPIs in a variety of contexts and at a variety of scales (Chen *et al.* 2023, Zhou *et al.* 2023, Will *et al.* 2023, Borghini *et al.* 2024, Edwards *et al.* 2024, Waksman *et al.* 2024, Kamal *et al.* 2025). However, the D-SCRIPT inference implementation only supports use of a single GPU, and PPI inference on large eukaryotic proteomes such as is required by the recent PHILHARMONIC pipeline (Sledzieski *et al.* 2025) requires up to hundreds of gigabytes of memory and several weeks.

We introduce Blocked, Multi-GPU Pairwise Inference (BMPI), which optimizes D-SCRIPT performance in memory-constrained and multi-GPU settings. D-SCRIPT with BMPI improves performance across the major classes of high-throughput D-SCRIPT tasks, with four modes: one for prediction on all pairs of proteins in an individual proteome; two different modes for predicting on specified pairs of proteins (depending on sparsity); and a specialized bipartite mode for prediction between proteins from *pairs* of proteomes. D-SCRIPT with BMPI allows a substantial reduction in memory usage, at a user-controlled level with a very small performance tradeoff, and enables multi-GPU parallelism (with only a small, fixed memory overhead) to speed prediction on large protein sets from weeks to days. We demonstrate these improvements in performance through varied experiments on metazoan proteins. Our improvements will improve the usability of D-SCRIPT for varied prediction tasks and levels of computational resources, e.g. enabling proteome-scale prediction to be performed on a single personal computer with limited memory or in greatly reduced time on a multi-GPU server or HPC environment. Note that the output of D-SCRIPT with BMPI is identical to the original; we only modify the order in which proteins are loaded and the set of requested pair predictions are computed by D-SCRIPT. Additionally, because it is agnostic to the prediction model, our implementation demonstrates a practical approach for up- or down-scaling the resource footprint of essentially arbitrary pairwise inference tasks, such as alternative deep PPI prediction methods (see Section 5).

## 2 Blocked multi-GPU pairwise inference

**Figure 1.**
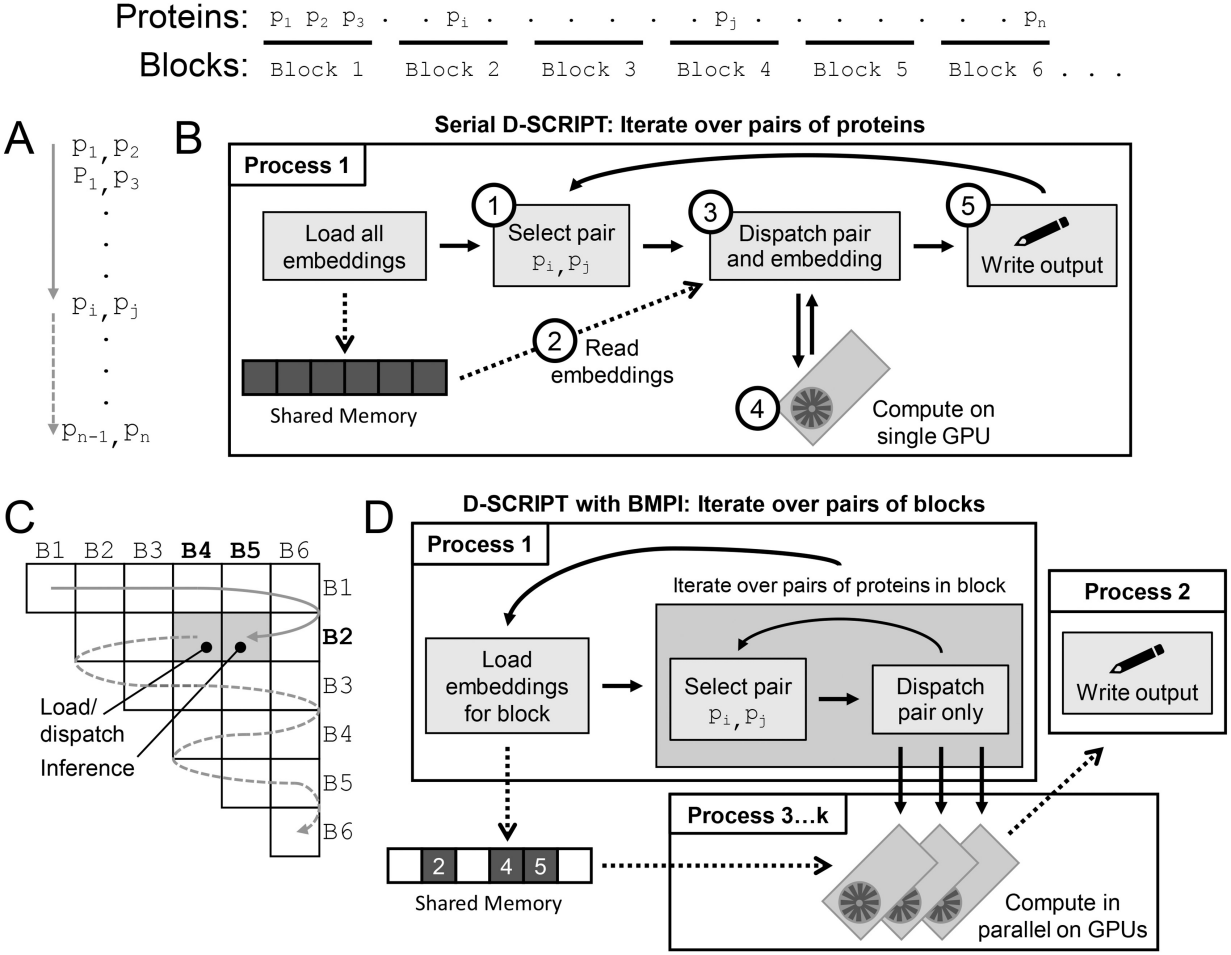
(A) The original serial version of D-SCRIPT iterates through each pair of candidate proteins. (B) This version uses only a single process, which pre-loads all protein embeddings into memory, then (1) iterates through each pair of proteins, (2) obtains the preloaded embedding from memory, (3) moves these embeddings into GPU memory and (4) makes a prediction, before finally (5) writing to the output file. (C) D-SCRIPT with BMPI iterates through pairs of blocks of proteins. Pre-embedded proteins are divided into a user-specified number of blocks (shown at top, with six blocks). The iteration through pairs of blocks (gray arrow) increments the first block (rows) but alternates incrementing/decrementing the second block (columns). So, consecutive pairs of blocks never require two new blocks and at most one new block is loaded into memory at a time, with the other block reused. Thus only three blocks of protein embeddings are required in memory at any time, significantly reducing the memory requirements of inference with relatively little runtime overhead. (D) The main process of D-SCRIPT with BMPI loads the embeddings for new blocks as required. For each pair of blocks, it iterates through pairs of proteins and submits them to a pool of GPUs each managed by their own processes. Each GPU process reads the necessary embeddings from shared memory and performs a forward pass of the model, enabling parallel PPI inference. Predictions are written to disk by a separate process, preventing inference from being stalled by relatively slow disk writes.

In the original D-SCRIPT implementation, inference proceeds serially: all protein embeddings are preloaded into memory (Supplementary Text, available as supplementary data at *Bioinformatics* online) and a single process iterates through all protein pairs, placing the corresponding embeddings onto the GPU for inference, and then retrieving and writing the output (Fig. 1A and B). Our new implementation, which is now the default behavior of D-SCRIPT, makes two advances (Fig. 1C and D). First, we separate (1) loading embeddings and iterating through proteins, (2) transferring data to/from a GPU, and (3) processing/writing output into separate processes (Fig. 1D). The main process handles (1), and enqueues references to pairs of protein embeddings loaded into shared memory. Multiple processes, each associated with a unique GPU, then dequeue inference tasks, transfer the embeddings to their GPU, and retrieve the model output. The outputs are passed, via a second queue, directly to a single writer process, which determines if predictions are above threshold and writes to output files accordingly. This approach allows for multiple GPUs to be employed in parallel and maximizes their utilization, preventing idling to wait for data loading or writing and sharing memory-intensive protein embeddings. Writing to output files is restricted to the single writer progress, which also preserves straightforward progress monitoring.

Our second advance is to divide the set of proteins into --blocks blocks. We then guarantee every protein pair will be examined by considering all pairs of proteins between all possible pairs of blocks (including self-pairs of the same block twice). Organizing the consideration of protein pairs in a block structure means we only need to have in memory embeddings of proteins from the blocks being considered (Fig. 1C and D). Using blocks, whose number (and therefore size) can be varied, achieves a tradeoff between preloading all embeddings (equivalent to one block) and just-in-time loading of individual protein embeddings, which would cause bottlenecks. The main process loads embeddings and prepares (enqueues) pairs of proteins for one pair of blocks while the GPUs are performing inference on the previous pairs. The iteration order through blocks is such that only one block of embeddings is loaded at a time, so—as loading is contingent on inference of previous pairs of blocks finishing—only three blocks’ worth of protein embeddings are needed in memory at any one time (Fig. 1C, Supplementary Text and Algorithm 1, available as supplementary data at *Bioinformatics* online).

The default behavior for D-SCRIPT with BMPI is to perform PPI inference on all pairs of proteins, as described above (a list of proteins is specified via --proteins). We also support predicting on only a subset of pairs; a user can instead provide a list of candidate pairs of proteins via --pairs. Invoking some-pairs prediction with one block is closest to the behavior of the original D-SCRIPT, with different internal representations (Supplementary Text, available as supplementary data at *Bioinformatics* online). In this second mode, the unique proteins from the specified pairs can still be broken into blocks; iteration through pairs of blocks proceeds as for complete inference, except that each pair of proteins is checked against the matrix before submission to the GPU. It is still the case that all protein embeddings are loaded for each block when each block is considered. We also implemented a third mode that is similar to the second mode but only loads selected proteins from each block into memory, based on the desired pairs of proteins for the current pair of blocks (invoked via the --sparse_loading flag, Supplementary Text, available as supplementary data at *Bioinformatics* online). Finally, we implemented a specialized inference mode for bipartite prediction between proteins from two sets/proteomes. This mode, invoked via the dscript predict_bipartite command, iterates through separate blocks from each species and can separately load proteins from each species (Supplementary Text, available as supplementary data at *Bioinformatics* online).

## 3 Benchmarking datasets

We obtained the fruit fly (*Drosopholia melanogaster*) proteome from FlyBase (FB2025_02 release) (Öztürk Çolak *et al.* 2024), and kept only the first protein isoform reported for each gene from the list of all translations. We then limited our analysis to proteins with a maximum length of 1000 amino acids, resulting in 12 563 proteins. First, we arbitrarily sampled half, to represent a small complete proteome (DMel50) with a size of 6282 proteins (19 728 621 pairs). Second, we randomly sampled 25% of possible pairs between all 12 563 proteins to represent a relatively “dense” set of pairs (DMel-pair25; 19 727 050 pairs). Third, to simulate prediction between two proteomes, we took all pairs of proteins from the *Wolbachia* symbiont of *D. melanogaster* (Holden *et al.* 1993) and *D. melanogaster* (wMel-DMel). We obtained 928 symbiont proteins with lengths between 100 and 1000 amino acids (inclusive) from RefSeq assembly GCF_016584425.1 (Goldfarb *et al.* 2025) and used the 12 563 fly proteins as above for 11 658 464 total pairs (13% of all possible pairs between proteins from either set). Finally, we used 38 486 protein sequences from the coral *Acropora millepora* (RefSeq assembly GCF_013753865.1), limited to a maximum length of 1200 amino acids. We formed a “sparse” pair set by sampling 3% of possible *Acropora* protein pairs (AMil-pair03, 22 217 005 pairs), where every protein appeared at least once in the set of pairs we sampled. All benchmarking used a local server equipped with 2 AMD EPYC 7502 processors, 2TB of RAM, and 8 NVIDIA A6000 GPUs (Supplementary Text, available as supplementary data at *Bioinformatics* online).

## 4 Performance improvements

We ran both our new D-SCRIPT inference implementation with BMPI and the original implementation across the four representative datasets described above, which cover both complete (all-pairs) and some-pairs inference use cases. We demonstrated D-SCRIPT with BMPI using multiple GPUs to reduce runtime, multiple protein blocks to reduce memory usage, and both together (Table 1). First, we ran our new implementation with all 8 GPUs but a single protein block. We observe an average speedup of 8.08×, which slightly exceeds the theoretical 8× speedup due to the cumulative effect of more minor modifications, e.g. writing output in parallel with inference. Using multiple GPUs has some memory overhead (below), so memory usage with 8 GPUs and one block is 15–30 GB higher than for the original implementation and one GPU.

**Table 1.** Observed runtime and memory usage for PPI inference with Original D-SCRIPT and with BMPI.^a^

Method	Dataset	GPUs	Blocks	Time	Mem.
Original	DMel50	1	—	52.06	61.10
BMPI-all	DMel50	8	1	6.42	87.97
BMPI-all	DMel50	1	64	50.91	8.39
BMPI-all	DMel50	4	64	12.82	19.48
BMPI-all	DMel50	8	64	6.62	34.55
Original	DMel-pair25	1	—	51.99	116.51
BMPI-some	DMel-pair25	8	1	6.45	145.95
BMPI-some	DMel-pair25	1	64	51.88	14.84
BMPI-some	DMel-pair25	8	64	6.74	39.19
Original	wMel-DMel	1	—	24.92	123.23
BMPI-some	wMel-DMel	8	1	3.07	150.25
BMPI-some	wMel-DMel	1	64	23.91	12.76
BMPI-some	wMel-DMel	8	64	3.36	37.80
Original	AMil-pair03	1	—	74.06	389.39
BMPI-some	AMil-pair03	8	1	9.24	419.90
BMPI-some	AMil-pair03	1	64	73.89	28.20
BMPI-some	AMil-pair03	8	64	9.87	55.08

a Selected parameters in all-pairs and some-pairs modes; sparse-loading and bipartite modes appear in the Supplementary Tables, available as supplementary data at *Bioinformatics* online. Datasets: DMel50, all pairs of 50% of *Drosophila melanogaster* proteins; DMel-pair 25, 25% of pairs of all *D. melanogaster* proteins; wMel-DMel, pairs of proteins between the *Wolbachia* symbiont and *D. melanogaster*; AMil-pair 03, 3% of pairs of all *Acropora millepora* proteins. Time, runtime in hours; Mem., estimated memory usage in gigabytes (GB).

Next, we ran blocked inference with a single GPU and 64 blocks. We observe dramatic reductions in peak memory usage (Table 1), e.g. from 61 GB to 8 GB for DMel50 and from 389GB to 28 GB for AMil-pair03. Thus, we reduce the memory requirement for prediction on a large proteome (e.g. *A. millipora*) from a high-memory server to a typical workstation. Memory savings increase with number of blocks, though they plateau at a point where memory usage is dominated by sources other than protein embeddings. (see Table 1, available as supplementary data at *Bioinformatics* online, for additional benchmarks with varying block sizes.) The runtimes are slightly faster than the original implementation; minor savings from our implementation include only loading one block of protein embeddings before inference begins. Using more, smaller blocks can carry a runtime cost, especially once the time required for loading embeddings (which scales linearly) exceeds the time taken by PPI inference (which scales quadratically).

We then ran blocked, multi-GPU parallel inference with 8 GPUs and 64 protein blocks. We observe that the runtimes are up to 10% slower than with a single block. We largely attribute this to the time required for loading embeddings approaching the time required for inference, leading to reduced GPU utilization. Consistent with this, smaller numbers of blocks (8–32) demonstrate less of a runtime increase compared to a single block, while further increasing the number of blocks can dramatically increase runtime (Table 1, available as supplementary data at *Bioinformatics* online). Across our datasets, we found 64 blocks to be a reasonable value where memory usage seems to plateau and the runtime penalty is still low. For larger numbers of pairs, with correspondingly faster-increasing PPI inference time, we would expect larger number of blocks to give reasonable performance as well (with further reduction in memory usage).

The memory usage observed with 8 GPUs and 64 blocks, e.g. 25 GB for DMel50 and 55 GB for AMil-pair03, is still far below the memory usage of the original implementation using a single GPU (Table 1), and well below what could be expected to be available on a multi-GPU machine. However, it is somewhat higher than memory usage with one GPU. By comparing corresponding 64-block runs, we can estimate that the overhead of each GPU (and associated process) is 3.66 GB, which is also consistent with the memory usage of a 4-GPU test performed with DMel50. For smaller protein sets (DMel50), additional GPUs can be the largest source of memory usage. So, if the goal is purely to minimize memory usage, using a single GPU may be preferred. Nonetheless, blocked, multi-GPU inference jointly provides strong runtime and memory usage improvements for large inference tasks.

Finally, we benchmarked our other two specialized inference modes, which both handle only specified protein pairs. We found that our “sparse-loading” mode consistently increases memory usage over our normal some-pairs mode even on our “sparser” datasets (Supplementary Text and Table 2, available as supplementary data at *Bioinformatics* online). However, it remains possible that in extreme cases this mode could offer some memory savings. We did observe a memory usage improvement in some cases when we benchmarked our bipartite mode (Table 3, available as supplementary data at *Bioinformatics* online) on our wMel-DMel dataset, which consists of pairs of proteins from a symbiont and its host (Supplementary Text, available as supplementary data at *Bioinformatics* online). But, we did not observe an improvement in runtime relative to our general implementation, treating all proteins as from one set (Table 3, available as supplementary data at *Bioinformatics* online). We expect that this is due to the runtime being dominated by the cost of PPI inference itself, which is the same for both modes. Nonetheless, bipartite mode may provide a simpler user experience as it can take as input separate embedding files and lists of proteins from each species rather than requiring generation of a single embedding file and a list of pairs.

## 5 Discussion

We have shown that BMPI substantially reduces the memory usage of and parallelizes D-SCRIPT inference. It enables proteome-wide PPI inference in less than a week: using 8 NVIDIA A100 GPUs, about 14 h for *D. melanogaster* and 6.5 days for the large *A. millipora* proteome. Our approach is quite generalizable: these improvements are entirely independent of the model underling D-SCRIPT’s PPI inference. Extending our approach to efficiently distribute computation at the level of pairs of blocks across a distributed compute cluster is a potential generalization left for future work. Our revised implementation represents a blueprint for efficiently parallelizing, and/or reducing the memory footprint of, any method based on pairwise prediction by a deep learning model. In particular, there are multiple alternative methods for deep-learning based PPI prediction (Bell *et al.* 2022, Szymborski and Emad 2022, Petrey *et al.* 2023, Soleymani *et al.* 2023, Ko *et al.* 2024, Ullanat *et al.* 2025), and a similar approach could also benefit them or future approaches.

## Supplementary Material

btaf564_Supplementary_Data

## Data Availability

Blocked, multi-GPU parallel inference is integrated into version 0.3 of the dscript command-line package, available from the PIP package repository (pip install dscript==0.3), on GitHub at https://github.com/samsledje/D-SCRIPT, or via Zenodo at https://doi.org/10.5281/zenodo.16325182 (archive of version 0.3.0). BMPI is now the default inference behavior of dscript predict; the previous inference procedure is still available as dscript predict_serial. Bipartite blocked inference on two protein sets (Supplementary Text, available as supplementary data at *Bioinformatics* online) is invoked via dscript predict_bipartite. The dependencies are unchanged. Usage requires that (a superset of) protein sequences be pre-embedded using dscript embed.
