# Supplementary material for: Memory-efficient, accelerated protein interaction inference with blocked, multi-GPU D-SCRIPT
Source: Bioinformatics. 2025 Oct 11;41(10):btaf564. doi: 10.1093/bioinformatics/btaf564 (PMC12553328; doi:10.1093/bioinformatics/btaf564)
Supplement: btaf564_Supplementary_Data [file btaf564_supplementary_data.pdf]

Supplementary Information for Memory-Efficient, Accelerated Protein Interaction Inference with Blocked, Multi-GPU D-SCRIPT

Daniel E. Schäffer, Samuel Sledzieski, Lenore Cowen, Bonnie Berger

1 Supplementary Text

1.1 Modifications to data embedding loading

Embeddings for PPI inference are read from an `h5` file in parallel via a process pool that returns to the main process. This is true for the original D-SCRIPT inference, but we modified the implementation a) to have persistent processes that each load many embeddings, as opposed to re-initializing the h5 file each time and b) to allow user control over the number of processes. It remains the case that the main process blocks to wait for the result of each round of data loading, rather than truly proceeding in the background. The revised implementation is partially shared by the existing (now `dscript predict_serial`) and new inference modes. All benchmarking runs used 16 loading processes, and loaded embeddings from a file containing all (length-filtered) proteins from the species of interest. For wMel-dMel, we used a single file containing embeddings of proteins from both species (*D. melanogaster* and its *Wolbachia* endosymbiont). The pre-embedding process is performed by `dscript embed`, which takes as input a FASTA file and an embedding model. It takes less time than the PPI inference, *e.g.,* 77 minutes for the 38,486 *A. millipora* proteins using an A6000 GPU.

1.2 Iteration order for protein blocks in BMGI

We include pseudocode (Algorithm S1) that shows our method for iteration through blocks of proteins. The iteration order for pairs of blocks is to first (outer) increment the first block i from 0 (to N_{blocks}), and then (inner) alternate—between outer iterations—incrementing the second block j from i (to N_blocks) or decrementing it (from N_blocks) to i. This ensures that subsequent pairs of blocks always have one block in common (see also Figure 1B), so only one block of embeddings needs to be loaded at a time. For example, suppose inference is ongoing for pairs of proteins drawn from blocks $(i, j-1)$, with i even. The main process would load embeddings for block j (using the loading pool, see above) and then enqueue pairs of proteins from blocks (i, j). Loading of the next block $(j+1)$ waits until the inference on the previous block pair $(i, j-1)$ is complete (see below), and embeddings are no longer required for the proteins from block $j-1$. So, only three blocks of protein embeddings are required in memory at any one time.

Completion of inference on a block pair is monitored by enqueuing into the GPU queue a special object containing an integer flag, after all pairs of proteins from that block pair. Once the last protein pair has been dequeued by a GPU process, the flag in the GPU queue is found and reported back by a GPU process. The main process tracks the current value of this flag, corresponding to the most recently enqueued pair, and waits for this flag to be reported before proceeding with the next loading (see Algorithm S1). Because only one GPU process dequeues/reports the flag, when using $N_{GPU} > 1$ GPUs , up to $(N_{GPU}-1)$ "old" protein embeddings may still be in use while loading the next block begins. Additionally, memory management (freeing of unneeded embeddings) is handled

implicitly by PyTorch, so actual usage may appear slightly higher on unconstrained systems. On the other hand, we incorporated special cases, such as for self-pairs of blocks (i, i), to remove redundant data loading when the needed embeddings are currently one of the three blocks in memory; these cases are excluded from the flag-waiting process described above (again, see Algorithm S1).

1.3 Internal representation of user-specified proteins and pairs

Our implementation represents each protein by an index, corresponding to its position in the input list. This index is used for efficient lookups of embeddings, and is the only identifier passed to the GPU processes. This also minimizes the amount of data that has to go through the inter-process queues. The output/writer process is initially provided with the list of protein names and maps indices (received from the GPUs) back to names.

For a list of pairs of proteins, our implementation extracts a list of unique proteins and creates a binary matrix indicating which pairs are to be predicted. This allows 1) trivially splitting the proteins into blocks using the list and 2) quickly looking up whether inference should be performed for a particular pair of proteins. In contrast, the original (serial) D-SCRIPT inference implementation uses a list of protein pairs, as strings, in all cases. Since the matrix representation has fixed size quadratic in the number of proteins, for very sparse pair sets, the storage required is asymptotically worse than the list of pairs stored by original D-SCRIPT. But, in practice binary elements are low-cost versus pairs of strings, and the memory required is much less than that for embeddings.

1.4 Sparse loading of proteins for some-pairs prediction increases memory utilization in benchmarks

When predicting on only some pairs, our implementation loads all embeddings for each block into memory—exactly as for all-pairs prediction—and only checks protein pairs against the binary matrix when determining which pairs to enqueue for the GPUs. In principle, this could waste memory when most protein pairs are skipped, as some proteins in a block could be unused for a particular pair of blocks. So, we implemented a different inference mode that instead, for each pair of blocks of proteins, first determines which proteins from each block are required for the specified list of pairs of interest. (This is done by checking for non-zero rows and columns in the sub-matrix corresponding to that pair of blocks.) Only those proteins' embeddings are loaded; addressing the limitation of the "dense" mode. But, as a consequence, it is not possible to share loaded embeddings between multiple pairs of blocks: if we are considering pairs of proteins between blocks (i, j) followed by pairs between blocks $(i, j+1)$, different subsets of block i might be required for each, so, for simplicity, all required embeddings for block i are (re-)loaded each time. So, while following the same principle of loading/submitting protein pairs from the "next" pair of blocks while inference is ongoing for the "previous" pair, the maximum memory usage is 4 block's worth instead of 3. This mode can be used with the `--sparse_loading` flag to `dscript predict` and is only available when more than one block is specified.

We benchmarked this mode on our our three some-pairs datasets, DMel-pair25 (25% of pairs), wMel-DMel (∼13% of pairs), and AMil-pair03 (3% of pairs). We observed consistently worse memory usage (Table S2, except for one test with WMel-DMel where memory usage was reduced by $< 2\%$. So for our datasets, any memory savings from embeddings not loaded are outweighed by other additional usage. We note the following tradeoff which may limit the memory savings across most cases in practice: For few, large blocks, the likelihood that a protein is included in zero pairs and can be skipped is low, so at most a small fraction of proteins (embeddings) can be skipped.

And for many, small blocks, it may be possible to skip a greater fraction of proteins from each block, but the total the size of (all embeddings for) each block is relatively low compared to other uses of memory, so the overall memory savings are again limited.

1.5 Bipartite prediction mode provides a convenient interface, but little to no performance benefit

Our implementation treats protein pairs as if they are arbitrarily selected from the set of all possible pairs. (And, the order of proteins used to form blocks is based on first occurrence in the list of pairs.) As a result, it may not have optimal memory or time usage when the choice of pairs is highly structured. In particular, we consider the case of pairs representing a bipartite graph, *i.e.*, one wants to predict all pairwise interactions between two separate protein sets. For example, one might want to predict PPIs between a set of effector proteins and a set of target proteins or between pathogen and host proteins. In this case, proteins from each set would be distributed among blocks by the modes above. If we consider dividing the two sets into a and b equal-sized blocks, respectively, we would need to consider all possible pairs of proteins $\binom{a+b}{2} = ab + \frac{1}{2}(a^2 + b^2 - a - b)$ pairs of blocks, predicting on only a subset of protein pairs from each. This creates unnecessary loading and preprocessing relative to a designed approach.

So, we added an additional prediction mode, `predict_bipartite` , specifically to make bipartite predictions. This mode takes *two* lists of proteins and corresponding embeddings (which may be combined or separated), along with (optionally) separate numbers of blocks into which each set is split. It then traverses through each pair of blocks drawn from the two sets, and predicts PPIs between all pairs of proteins between the two blocks. Like the first two modes, the traversal order increments the first block and alternates the direction of each second block to be able to reuse embeddings in memory. But, because the sets of first and second blocks are disjoint (rather than the same), each first block must be paired with every second block, not just higher-numbered ones. Still, by separating the two sets of proteins we need to consider only ab pairs of blocks, in contrast to above. The maximum memory usage is still three blocks' worth of embeddings; with two blocks of the second set during the inner traversal and two blocks of the first set when incrementing the outer traversal.

1.6 Benchmarking Methodology

We used the original D-SCRIPT inference model (DOI: `doi.org/10.57967/hf/6441`) for all benchmarks. Runtimes were measured with the "real" output of the bash `time` utility. To conservatively measure memory usage across all processes, we recorded the value of "MemAvailable" from `/proc/meminfo` approximately once per minute during each run and compared the minimum value to the value immediately before starting. We chose this approach to make sure that memory usage of all components were accounted for, including any processes which are detached from the main process group (*e.g.*, PyTorch shared memory management processes). We expect that reported memory usages are generally slight overestimates because of unrelated fluctuations in system memory usage; as an approximate upper bound, we observed variation of $\sim$2.9GB in initial available memory values across our benchmarks.

We provide scripts used for generating datasets (described in the main text) as well as for memory monitoring in the GitHub repository, in `scripts/bmpi_bench`.

2 Supplementary Materials

Method	Dataset	GPUs	Blocks	Time (h)	Memory (GB)
BMPI-all	DMel50	8	1	6.42	87.97
BMPI-all	DMel50	8	8	6.48	52.93
BMPI-all	DMel50	8	16	6.53	42.40
BMPI-all	DMel50	8	32	6.56	37.30
BMPI-all	DMel50	8	64	6.62	34.55
BMPI-some	wMel-DMel	8	1	3.07	150.25
BMPI-some	wMel-DMel	8	8	3.11	76.80
BMPI-some	wMel-DMel	8	16	3.16	55.38
BMPI-some	wMel-DMel	8	32	3.24	41.92
BMPI-some	wMel-DMel	8	64	3.36	37.80
BMPI-some	AMil-pair03	8	1	9.24	419.90
BMPI-some	AMil-pair03	8	8	9.15	179.81
BMPI-some	AMil-pair03	8	16	9.25	109.17
BMPI-some	AMil-pair03	8	64	9.87	55.08
BMPI-some	AMil-pair03	8	256	11.63	40.52

Table S1: We performed several additional tests of D-SCRIPT with BMPI (in all-pairs and some-pairs modes) with additional numbers of blocks on three datasets. Results with 1 and 64 blocks are reproduced here from Table 1. We used 8 GPUs for all of these benchmarks to reduce the time required; one can estimate the single-GPU memory usage by subtracting the difference between 1- and 8-GPU benchmarks reported in Table 1, *ca.* 26GB. (Of course, the time taken would increase by $7.5\times - 8\times$.) Datasets: DMel50, all pairs of 50% of *Drosophila melanogaster* proteins; wMel-DMel, pairs of proteins between the *Wolbachia* symbiont and *D. mel.*; AMil-pair 03, 3% of pairs of all *Acropora millepora* proteins.

Method	Dataset	GPUs	Blocks	Time (h)	Memory (GB)
BMPI-some	DMel-pair25	16	8	6.53	54.86
BMPI-sparse	DMel-pair25	16	8	6.46	60.73
BMPI-some	wMel-DMel	16	8	3.16	55.38
BMPI-sparse	wMel-DMel	16	8	3.13	54.41
BMPI-some	wMel-DMel	8	8	3.36	37.80
BMPI-sparse	wMel-DMel	64	8	3.14	39.80
BMPI-some	AMil-pair03	16	8	9.25	109.17
BMPI-sparse	AMil-pair03	16	8	9.27	131.30
BMPI-some	AMil-pair03	64	8	9.87	55.08
BMPI-sparse	AMil-pair03	64	8	9.94	59.38
BMPI-some	AMil-pair03	256	8	11.63	40.52
BMPI-sparse	AMil-pair03	256	8	12.03	41.50

Table S2: We compared the normal some-pairs mode of D-SCRIPT with BMPI to a modified implementation ("sparse") that skips loading unneeded protein embeddings from each block using selected numbers of blocks across three some-pairs datasets. Non-sparse results with wMel-DMel and AMil-pair03 are reproduced here from Table S1. Datasets: DMel-pair 25, 25% of pairs of all *Drosophila melanogaster* proteins; wMel-DMel, pairs of proteins between the *Wolbachia* symbiont and *D. mel.*; AMil-pair03, 3% of pairs of all *Acropora millepora* proteins.

Method	Dataset	GPUs	Blocks	Time (h)	Memory (GB)
BMPI-some	wMel-DMel	1	8	3.07	150.25
BMPI-bipar	wMel-DMel	1, 1	8	3.48	147.86
BMPI-some	wMel-DMel	4	8	3.08	123.59
BMPI-bipar	wMel-DMel	1, 4	8	3.40	95.98
BMPI-some	wMel-DMel	8	8	3.11	76.80
BMPI-bipar	wMel-DMel	1, 8	8	3.40	66.59
BMPI-some	wMel-DMel	16	8	3.16	55.38
BMPI-bipar	wMel-DMel	1, 16	8	3.37	51.77
BMPI-some	wMel-DMel	32	8	3.24	41.92
BMPI-bipar	wMel-DMel	1, 32	8	3.33	44.20
BMPI-some	wMel-DMel	64	8	3.36	37.80
BMPI-bipar	wMel-DMel	1, 64	8	3.33	41.02

Table S3: We compared the normal some-pairs mode of D-SCRIPT with BMPI to a modified implementation ("bipar") that iterates through blocks of proteins from two species (or sets) and predicts cross-species protein interactions. The wMel-DMel dataset consists of pairs of proteins with one each from the *Wolbachia* bacterial endosymbiont (wMel) of *Drosophila melanogaster* and from *Drosophila* (DMel). For the bipartite mode, blocks are reported as (# used for wMel), (# used for DMel); the former was always 1. Non-bipartite results, which combined the proteins from both organisms into one set, are reproduced here from Table S1.

Algorithm S1 Blocked Protein Iteration for BMPI

```
1: blocks ← number of protein blocks to use
2: Embeds_1, Embeds_2, Embeds_3 ← LOAD(0), ∅, ∅        ▷ Each references to one blocks' embeddings
3: cp ← 0                  ▷ Flag associated with current block pair to await before next data loading
4: for i ∈ [0, blocks) do                                                        ▷ Outer iteration
5:     if i ≡ 0  mod 2 then
6:         SUBMIT_SELF(i, Embeds_1)                                  ▷ Self-pair submission (i = j)
7:         if i = 0 and blocks > 1 then Embeds_3 ← LOAD(1) end if
8:         for j ∈ [i + 1, blocks) do                                 ▷ Incrementing inner iteration
9:             if j = i + 1 then
10:                Embeds_2 ← Embeds_3                                 ▷ Reuse previously-loaded data
11:                if j ≠ blocks − 2 then Embeds_3 = ∅ end if
12:            else
13:                Embeds_2 ← LOAD(j, flag : (cp − 1))
14:            end if
15:            cp ← cp + 1
16:            SUBMIT_PAIR(i, j, Embeds_1, Embeds_2, flag : cp)
17:        end for
18:    else
19:        if i = blocks − 1 then   ▷ Special case to reuse loaded embeddings in the final iteration
20:            Embeds_1 ← Embeds_2
21:        else if i = blocks − 2 then
22:            Embeds_1 ← Embeds_3
23:        else
24:            Embeds_1 ← LOAD(i, flag : (cp − 1))
25:        end if
26:        for j ∈ [blocks − 1, i + 2) do                             ▷ Decrementing inner iteration
27:            cp ← cp + 1
28:            SUBMIT_PAIR(i, j, Embeds_1, Embeds_2, flag : cp)
29:            Embeds_2 ← LOAD(j − 1, flag : (cp − 1))
30:        end for
31:        if i < blocks − 2 then                      ▷ Special cases to re-use loaded embeddings
32:            SUBMIT_PAIR(i, i + 2, Embeds_1, Embeds_2)
33:            cp ← cp + 1
34:            Embeds_3 ← Embeds_2
35:        end if
36:        SUBMIT_SELF(i, Embeds_1)          ▷ Self-pair submission (i = j) using loaded embeddings
37:        if i < blocks − 1 then         ▷ Special case; after (36) to balance loading & submission
38:            if i ≠ blocks − 2 then emebds_2 ← LOAD(i + 1, flag : cp + 1) end if
39:            cp ← cp + 1
40:            SUBMIT_PAIR(i, i + 1, Embeds_1, Embeds_2, flag : cp)
41:        end if
42:    end if
43: end for
```

```
Proteins ← List of protein names
N ← length(Proteins)
N_block ← ⌈N/blocks⌉

procedure BOUNDS(b)
    return b * N_block , min((b + 1) * n_block, N)
end procedure

flagQ ← Queue to receive indications of completed block pairs from GPUs
procedure LOAD(b, flag : 0)
    if flag > 0 then
        last ← GET(flagQ)
        while last ≠ flag do        ▷ Occurs occasionally with multiple GPUs,
                                    ▷ when tasks may be completed slightly out of order
            last′ ← GET(flagQ)
            PUT(flagQ)
            last ← last′
        end while
    end if
    return Embedding data for Proteins[BOUNDS(b)]
end procedure

gpuQ ← Queue to pass inference tasks to GPUs
procedure SUBMIT_SELF(b, E)
    for pairs of proteins p1, p2 ∈ Proteins[BOUNDS(b)] do
        PUT(gpuQ, (p1, p2, E[p1], E[p2]))        ▷ p1 and p2 are indices identifying proteins and
                                                 ▷ E[p1] and E[p2] are the corresponding embeddings.
    end for
end procedure

procedure SUBMIT_PAIR(b1, b2, E1, E2, flag : 0)
    for proteins p1 ∈ Proteins[BOUNDS(b)1] do
        for proteins p2 ∈ Proteins[BOUNDS(b)2] do
            PUT(gpuQ, (p1, p2, E1[p1], E2[p2]))        ▷ Again, referenced by index
        end for
    end for
    if flag > 0 then PUT(gpuQ, "end of pair flag") end if
end procedure
```
